# NOX Dependent ROS Generation and Cell Metabolism

**DOI:** 10.3390/ijms24032086

**Published:** 2023-01-20

**Authors:** Tiziana Pecchillo Cimmino, Rosario Ammendola, Fabio Cattaneo, Gabriella Esposito

**Affiliations:** 1 Department of Molecular Medicine and Medical Biotechnology, School of Medicine, University of Naples Federico II, 80131 Naples, Italy; 2 CEINGE Advanced Biotechnologies Franco Salvatore S.c.a.r.l., 80131 Naples, Italy

**Keywords:** NADPH oxidase, NOX, reactive oxygen species, ROS, cell metabolism, redox metabolism, metabolic reprogramming, glycolytic enzymes

## Abstract

Reactive oxygen species (ROS) represent a group of high reactive molecules with dualistic natures since they can induce cytotoxicity or regulate cellular physiology. Among the ROS, the superoxide anion radical (O2·−) is a key redox signaling molecule prominently generated by the NADPH oxidase (NOX) enzyme family and by the mitochondrial electron transport chain. Notably, altered redox balance and deregulated redox signaling are recognized hallmarks of cancer and are involved in malignant progression and resistance to drugs treatment. Since oxidative stress and metabolism of cancer cells are strictly intertwined, in this review, we focus on the emerging roles of NOX enzymes as important modulators of metabolic reprogramming in cancer. The NOX family includes seven isoforms with different activation mechanisms, widely expressed in several tissues. In particular, we dissect the contribute of NOX1, NOX2, and NOX4 enzymes in the modulation of cellular metabolism and highlight their potential role as a new therapeutic target for tumor metabolism rewiring.

## 1. Introduction

Reactive oxygen species (ROS) are generated from several cell types and include a group of molecules derived from the reduction of molecular oxygen. Based on magnitude, duration, and the site of generation, ROS show a dualistic nature since they can induce cytotoxicity or regulate cellular physiology. A detrimental effect is observed at high concentrations, whereas at low concentrations they function as second messengers, acting as regulators of cellular signaling [1]. Among ROS, the superoxide anion radical (O2·−) is a key redox signaling molecule, generated prominently by members of the NADPH oxidase (NOX) enzyme family and by the mitochondrial electron transport chain [2,3].

The NOX family represents the main source of controlled ROS formation and includes seven isoforms with a broad tissue distribution and activation mechanism [3]. Their subcellular distribution varies in the different cell types, ranging from plasma membrane to intracellular compartments and nuclear membrane [4,5]. The NOX family includes NOX1, NOX2, NOX3, NOX4, NOX5, and the dual oxidase Duox1 and Duox2. NOX2 is the first isoform characterized and consists of at least six different subunits, whose interaction is required to form an active enzymatic complex [6]. In unstimulated conditions, the two integral membrane proteins gp91^phox^ and 22^phox^ (the heterodimeric cyt b558) do not interact with the cytosolic regulatory subunits, p40^phox^, p47^phox^, p67^phox^, and Rac1/2 [7]. Upon stimulation, p47^phox^ undergoes rapid phosphorylations and, in turn, interacts with p67^phox^ triggering a membrane translocation of the cytosolic subunits and their interaction with the membrane cyt b558 to form the active oxidase complex able to generate superoxide anion. The activated NOX complex transfers electrons from the substrate (NADPH) to molecular oxygen through a prosthetic group (flavin) and heme group(s). NOX1 and NOX3 are regulated through a similar molecular mechanism, whereas Nox5, Duox1, and Duox2 are activated by receptor-linked stimuli that elevate cellular calcium levels [8,9]. NOX activation is finally regulated in order to prevent ROS overproduction, with the exception of NOX4 that does not require any further cytosolic subunits and therefore is constitutively active [10].

In phagocytic leukocytes, NOX-dependent superoxide generation plays a crucial role in destroying phagocytosed organisms and facilitating the anti-microbial function of cells [11], whereas in most cells and tissues NOX-dependent ROS production is implicated in biosignaling and pathophysiological functions [12], such as cardiovascular [13,14], neurodegenerative [15,16], cancer [17,18], and metabolic [19,20] diseases.

The specific effects of ROS are mainly associated with the covalent modification of specific cysteine residues localized within redox-sensitive target proteins. As a consequence of the oxidation of these specific and reactive cysteine residues, the activity of protein tyrosine phosphates (PTPs), as well as of many enzymes, are reversibly modified [21], thus promoting the phosphorylation of cytosolic residues of tyrosine kinase receptors (TKRs) [22,23,24,25] and serine/threonine kinase receptors (RSTK) [26,27]. These trigger, in turn, the activation of intracellular signaling proteins involved in several cell functions, such as metabolism, proliferation, and oxidative stress responses [28,29,30].

Oxidative stress and the cellular metabolism of cancer cells are strictly intertwined. In fact, high ROS levels, derived by metabolic and microenvironment-associated alterations, contribute to the modulation of cancer cell metabolism [1] and NOX enzymes play a key role in this process. Furthermore, an altered redox balance and deregulated redox signaling are recognized as hallmarks of cancer and are involved in malignant progression and resistance to drugs treatment.

The interplay of NOX-modulated redox signaling pathways associated with metabolism is still far from being fully understood. A better understanding of how NOX enzymes drive these complex adaptive responses will provide new mechanistic insights into metabolic reprogramming and may contribute to the development of novel therapeutic strategies.

In this review, we focus on the emerging roles of the NOX family as important modulators of metabolic reprogramming. Currently, only NOX1, NOX2, and NOX4 isoforms have been reported as implicated in this process. Therefore, we discuss the discuss the contribute of NOX1, NOX2, and NOX4 enzymes in the modulation of cellular metabolism and highlight their potential role for new therapeutic approaches that target the rewired metabolism of cancer cells.

## 2. NOX-Dependent Regulation of Cellular Metabolism

Increasing evidence indicates the critical role of NOX in the regulation of glucose, lipid, nucleotide, and protein metabolism, as well as in the metabolic reprogramming of cancer cells [31,32]. The most explored metabolic effects exerted by ROS generated by NOXs are associated with glucose metabolism. For instance, in PCB118-stimulated hepatocellular carcinoma cells, an enhanced aerobic glycolysis, lactate production, GLUT1, lactate dehydrogenase (LDHA), pyruvate dehydrogenase kinase (PDK), and pyruvate kinase M2 (PKM2) expression is observed [33]. ROS scavengers or NOX inhibitors significantly suppress PCB118-induced glucose consumption, lactate production, and aerobic glycolysis-related gene expression, thereby supporting the key role of NOX-dependent ROS generation in the glucose metabolism reprogramming of these cells [33]. Glucose is an essential source of energy for supporting all mammalian life and its metabolism involves multiple processes, including glycolysis, gluconeogenesis, pentose phosphate pathway (PPP), glycogenolysis, and glycogenesis. In anaerobic conditions, glycolysis produces lactate, whereas CO_2_ is generated in mitochondria upon full oxidation of glucose via respiration in aerobic conditions. Tumor or proliferating cells show an increased uptake of glucose that is metabolized in lactate in the aerobic glycolysis, even in the presence of oxygen and fully functioning mitochondria.

A critical factor involved in glucose metabolic reprogramming is hypoxic inducible factor 1 α (HIF1α) [34]. Some evidence demonstrates that ROS released by NOX contribute to metabolic reprogramming by stabilizing HIF1α, for instance in hypoxia-stimulated HUVE cells [35]. Here, HIF1α stabilization requires the activation of PKC and PI3K signaling and is accompanied by augmented hexokinase activity and membrane translocation of GLUT1. Interestingly, ROS scavenging or NOX inhibition completely reverts hypoxia-induced HIF1α accumulation and hexokinase activity, suggesting that ROS production is upstream of HIF1α signaling.

Glucose and glutamine play a key role in the metabolic reprogramming of cancer cells and represent the primary sources of carbon atoms for the biosynthesis of several molecules. In particular, glutamine is an important nitrogen donor for the production of nucleotides, amino acids, and nicotinamide. Carbamoyl-phosphate synthetase 2, aspartate transcarbamylase, and dihydroorotase (CAD) form a multifunctional enzyme that regulates de novo synthesis of pyrimidine nucleotides. In mammals, CAD is phosphorylated at the Ser1859 by S6 kinase 1 (S6K1), a downstream ribosomal protein target of mTORC1, thus stimulating the first three steps of the pyrimidine synthesis and allowing the cells’ overall progression through S phase of the cell cycle. S6K1 activity is finely regulated by redox-sensitive mechanisms that control its phosphorylation, its interaction with mTORC1, and the kinase activity of the S6K1-mTORC1 complex.

Interestingly, in several cell types, formyl peptide receptor 2 (FPR2) induces NADPH oxidase activity [23,24,28,29,36,37,38,39]. We analyzed, in human CaLu-6 cells, the ability of NOX-dependent ROS production to regulate CAD phosphorylation at the Ser1859 residue. The blocking of NOX activity by preincubation with apocynin or by Crispr/Cas technique completely prevents FPR2-induced CAD phosphorylation/activation, thereby revealing that NOX plays a crucial role in the metabolic reprogramming of anaplastic lung cancer cells by redirecting glutamine into anabolic pathways [40].

NOX-dependent ROS generation also contributes to the progression of metabolic diseases such as metabolic syndrome, obesity, and type 2 diabetes [41], as observed in a mouse cell line and in human skeletal muscle primary cells. The stimulation of these cells increases NOX-dependent production of ROS and triggers a shift towards a more glycolytic phenotype, which is sensitive to antioxidants and NOX inhibition, rather than to mitochondrial respiration [42].

## 3. NOX1 and the Modulation of Cellular Metabolism

NOX1 was the first homolog of NOX2 and was initially named mitogenic oxidase 1 (mox-1) [43] or NADPH oxidase homolog 1 (NOH-1) [44]. NOX1 and NOX3, the second NOX cloned isoform, share 60% sequence identity with NOX2 and for this reason are considered the closest isoforms to phagocytic NADPH oxidase. NOX1 isoform is expressed in a variety of tissues [45] but it is predominant in colon, prostate, and vascular cells [46]. Its expression can be induced by many conditions [45]. NOX1 activation requires the presence of the cytosolic subunit NOXO1 and NOXA1, the membrane subunit p22^phox^, and the small GTPase Rac. ROS generated via NOX1 are involved in several physiological processes implicated in cell metabolism regulation (Table 1).

**Figure 1:**
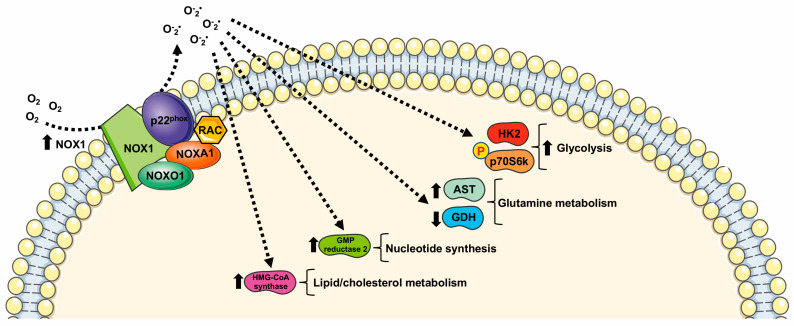
Metabolic pathways supported by NOX1 expression and NOX1-related ROS generation. HK2: Hexokinase II; GDH: glutamate dehydrogenase; AST: aspartate aminotransferase; HMG-CoA synthase: hydroxymethylglutaryl-CoA synthase.

However, aberrant NOX1 activation and/or expression is involved in a growing number of diseases, including neurological disorders, atherosclerosis, hypertension, inflammation, and cancer [45], through the deregulation of cell metabolism (Figure 1).

Indeed, NOX1 is upregulated in several tumors, where it functions as an oncogene [31,48]. Such upregulation is critical for elevated glycolysis and provides additional NAD^+^ in cancer cells with mitochondrial dysfunction [48,49,50]. NOX1 upregulation is also observed in cancer cells with compromised mitochondria functions due to the activation of oncogenic Ras or the loss of p53, and in primary pancreatic cancer tissues. The blocking of NOX1 functions selectively impairs cancer cells with mitochondrial dysfunction, leading to a decrease in cellular glycolysis, a loss of cell viability, and an inhibition of cancer growth in vivo, suggesting that NOX1 is a potential novel target for cancer treatment [31].

NOX1 is expressed in pancreatic β-cells [51] and plays an important role for ROS production during glucose-stimulated insulin secretion [52]. A further connection between the glucose-sensing mechanism and NOX1 is observed in C2C12 cells that show a NOX1-dependent glucose sensing pathway for selective phosphorylation of p70S6k. This kinase is involved in many cellular functions including protein synthesis, cell growth, cell cycle progression, and the regulation of insulin signaling. Interestingly, insulin-stimulated phosphorylation of p70S6k depends on both glucose and NOX1 [47].

NOX1-dependent metabolic rewiring is not limited to glucose oxidation through glycolysis. In hepatoma cells, NOX1 silencing induces a decrease in mitochondrial phosphoenolpyruvate carboxykinase (PEPCK-M), which converts oxaloacetic acid to phosphoenolpyruvate (PEP), thus re-channeling the mitochondrial intermediates of the tricarboxylic acid cycle (TCA) into the cytosolic pool of glycolytic intermediates. PEP can be directed towards the PPP for NADPH generation and nucleotide synthesis [32]. NOX1 also induces an increase in UTP-glucose-1-phosphate uridylyltransferase, suggesting an involvement of NOX1 in modulating glycogen biosynthesis [32].

NOX1 levels are also inversely correlated to the levels of mitochondrial glutamate dehydrogenase and positively correlated with aspartate aminotransferase, thus playing a role in the reprogramming of glutamine metabolisms. Moreover, NOX1 expression correlates with the amount of GMP reductase 2 modulating, in turn, nucleotide synthesis. Finally, reduced NOX1 levels result in a reduced expression of cytosolic hydroxymethylglutaryl-CoA synthase (HMG-CoA synthase). Thus, NOX1 may contribute to remodeling lipid metabolism in tumor cells since HMG-CoA synthase catalyzes the second step of the mevalonate biosynthesis, leading to lipid, steroid (including cholesterol), and isoprenoid biosynthesis [32].

## 4. NOX2 and the Rewiring of Metabolism

NOX2, also known as gp91^phox^, represents the first discovered member of the NOX family [45]. It is highly expressed in monocytes, macrophages, and granulocytes where it contributes to innate immune response [53]. However, NOX2 expression has also been observed in several tissues and nonphagocytic cells including neurons, hepatocytes, hematopoietic stem cells, endothelial cells, cardiomyocytes, and skeletal muscle myocytes [54]. NOX2 is constitutively associated with the protein p22^phox^ and its activation requires translocation of cytosolic factors to NOX2/p22^phox^ complex. Phosphorylation of the cytosolic subunit p47^phox^ is crucial for the recruitment and translocation of the other cytosolic subunits, p40^phox^, p67^phox^, and Rac. Subcellular NOX2 localization depends on the specific cell type. In phagocytic and other nonphagocytic cells, it shows mainly an intracellular and plasma membrane localization. However, in smooth muscle and endothelial cells NOX2 results localized in perinuclear cytoskeleton [5,55,56]. Enhanced NOX2-derived ROS production is responsible for an oxidized microenvironment that impacts deeply on tumorigenesis, tumor progression, cell proliferation [57], and cell metabolism [58] (Table 2).

**Figure 2:**
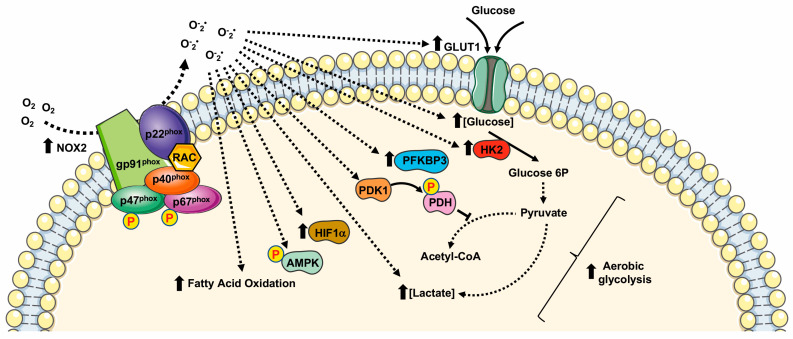
Metabolic pathways supported by NOX2 expression and NOX2-related ROS generation. HIF1α: Hypoxic Inducible Factor 1 α; PDK1: pyruvate dehydrogenase kinase 1; PDH: pyruvate dehydrogenase; HK2: Hexokinase II; PFKFB3: 6-phosphofructo-2-kinase/fructose-2,6-bisphosphatase.

Many cancer-related events undergo to a modified redox state, such as the inactivation of PTPs by oxidation of their thiol groups [13,27,30,66], DNA damaging [67], genomic instability [68], the regulation of transcriptional factors [69,70], and the modulation of cell metabolism [40,71] (Figure 2).

Several cellular systems that neutralize ROS are induced in an oxidative microenvironment. Among them, the transcription factor Nrf2 crucially regulates cellular antioxidative enzymes production. In resting conditions, binding to Keap1 inhibits Nrf2 activity. Upon oxidation of Keap1 cysteine residues by NOX2-derived ROS, Nrf2 is released and translocates to the nucleus where it binds to antioxidant response elements [57].

Hypoxia is a peculiarity of the tumor microenvironment that activates the hypoxia-inducible factor (HIF) family of transcription factors. HIFs, by mediating cellular adaption to low oxygen levels may influence several aspects of cancer. NOX2-dependent ROS generation induces the activation of HIF1α and thereby stimulates HIF-related cancer events [57].

Acute hypoxia induces anaerobic glycolysis to compensate cellular energy demands, and NOX2 activation is an early and crucial driver of a cascade of metabolic changes that promote glycolysis. In fact, NOX2-mediated stabilization of HIF1α contributes to anaerobic glycolysis by the direct activation of PDK1, which inactivates pyruvate dehydrogenase. This is one of the enzymes that form the pyruvate dehydrogenase complex (PDC) that catalyzes the conversion of pyruvate into acetyl-CoA. Thus, pyruvate is not directed to TCA and is converted into lactate, leading to the attenuation of mitochondrial respiration [58,59].

Similarly, in ovarian cancer cells and in RAW264.7 monocyte/macrophage-like cells, NOX2-dependent HIF1α activation induces glycolysis by enhancing the expression of GLUT1 and of the glycolytic enzyme hexokinase [60,61]. Furthermore, the high levels of NOX2 observed in patients affected by glioblastoma multiforme (GBM) correlate with high levels of hexokinase 2 and glucose uptake [64].

Although NOX2-mediated ROS generation promotes glucose oxidation, a metabolic-dependent feedback loop allows glycolysis to increase the expression of NOX2 and p47^phox^ to improve the ability of macrophages to produce ROS. In these cells, the resulting induction of the metabolic switch from oxidative phosphorylation to glycolysis results in an increase in NADPH levels, the major electron donor for NOX2 activity, also suggesting the activation of PPP [72].

NOX2 is the primary source of superoxide in primary acute myeloid leukemia (AML) cells. In these cells NOX2-dependent ROS generation promotes an increase in the uncoupling protein 2 (UCP2) and phosphorylation of AMPK, thus upregulating the expression of 6-phosphofructo-2-kinase/fructose-2,6-bisphosphatase (PFKFB3), a key regulatory glycolytic enzyme. Overexpression of PFKFB3 promotes glucose uptake and cell proliferation, whereas downregulation of PFKFB3 strongly suppresses leukemia growth both in vitro and in vivo [62].

In AML patient blasts, NOX2-derived ROS are also implicated in sphingolipid metabolism, fatty acid oxidation (FAO), purine metabolism, and amino acid homeostasis. In fact, the blocking of NOX2 functions results in an alteration of sphingosine and sphinganine levels, as well as in the inhibition of the transport of FAO. The inhibition or silencing of NOX2 results in the alteration of purine metabolism and amino acid homeostasis [63].

Cytosolic subunits of NOX2 constitute a complex with the phosphorylated form of 6-phosphofructo-2-kinase (PFK-2), in stimulated neutrophils. Phospho-PFK-2 catalyzes the production of fructose-2,6-bisphosphate, which is the main allosteric activator of phosphofructo-1-kinase, the limiting enzyme in glycolysis. The silencing or pharmacological inhibition of PFK-2 results in a significant reduction of NOX2 activity in neutrophils and, consequently, of the glycolytic rate. Therefore, modulation of NOX2 activity in neutrophils affects the glycolysis rate, highlighting the role of NOX2 in supporting the increase in energy metabolism, not only that of ROS production [65].

## 5. NOX4

NOX4 is considered the most evolutionarily distant NOX homolog sharing only 39% of homology with NOX2 [54]. NOX4 shows four splice variants and its functional expression, as well as its physiopathological role, has been described in several tissues and cell types [73,74,75,76,77,78,79,80,81,82].

ROS-generation by NOX4 depends on its interaction with p22^phox^, but does not require the recruitment of cytosolic subunits and Rac [83]. Therefore, NOX4 is considered a constitutively active enzyme, mainly regulated at the transcriptional/translational levels [84]. NOX4 expression is upregulated in oxidative injury [85] and upon TGF-β1 [86], TNF-α [87], and angiotensin-II [88] stimulation. NOX4-mediated ROS production can be induced by hypoxia [89] and by stimulation with lipopolysaccharide [90], insulin [91], and angiotensin [92].

**Figure 3:**
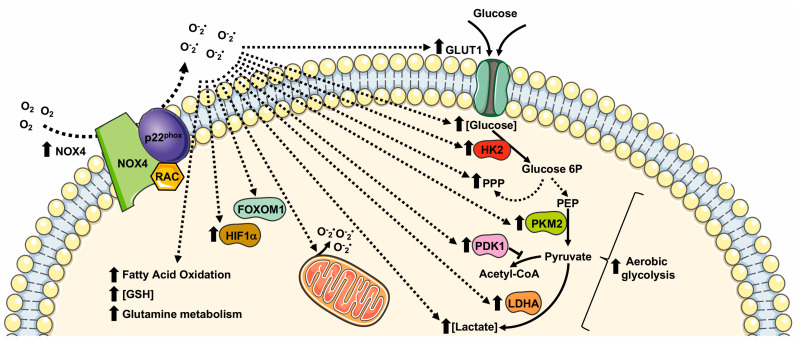
Metabolic pathways supported by NOX4 expression and NOX4-related ROS generation. GLUT1: glucose transporter 1; LDHA: lactate dehydrogenase A; PKM2: pyruvate kinase M2; PPP: pentose phosphate pathway; GSH: glutathione; FOXM1: Forkhead box M1; HIF1α: Hypoxic Inducible Factor 1 α; HK2: Hexokinase II; PDK1: pyruvate dehydrogenase kinase-1.

NOX4 is localized in mitochondria [93], endoplasmic reticulum (ER) [94], focal adhesion [95], nuclei [96], and it is associated with actin network [97]. Coherently with its pleiotropic localization, NOX4 is involved in the regulation of ER stress, DNA damage, the modification of extra cellular matrix (ECM), and mitochondrial ROS (mtROS) production, as well as cell tonicity and motility [18]. NOX4-derived ROS control a variety of cellular processes linked to cell proliferation, migration, survival, transformation, cell metabolism, and metabolic reprogramming (Figure 3) in different physiopathological processes in several tissues and cell types (Table 3).

NOX4 directs glucose metabolism not only toward glycolysis but also to PPP for the production of NADPH in non-small cell lung cancer (NSCLC) cells. NOX4 also supports glutamine metabolism for GSH production via ROS/PI3K/Akt signaling, thus contributing to the oxidative adaption of these cells [98]. NOX4 silencing significantly reduces GLUT1, LDHA, and PKM2 expression, and NOX4-deficient cells display an impaired glycolytic phenotype characterized by decreased ATP production, glucose consumption, lactate production, and NADPH generation [98].

Similarly to NOX2, NOX4-derived ROS are involved in HIFα activation. In glioblastoma, the aberrant NOX4-dependent ROS generation affects the regulation of FOXM1 by mediating HIF1α stabilization. Overexpression of NOX4 or FOXM1 promotes aerobic glycolysis, whereas the knockdown of NOX4 or FOXM1 significantly suppresses aerobic glycolysis [99]. TGF-β1 mediates NOX4 upregulation that, in turn, promotes ROS generation, growth, survival, hypoxia, and the angiogenesis of glioblastoma [100,101]. TGF-β1 is also required for the NOX4-dependent stabilization of HIF1α and of its nuclear accumulation, which results in metabolic reprogramming and in promoting the epithelial mesenchymal transition (EMT) of glioblastoma. TGF-β1 stimulation induces glycolysis and reduces mitochondrial respiratory capacity by increasing the protein expression levels of GLUT1, hexokinase-2 (HK2), LDHA, and PDK1 [102].

NOX4-dependent HIFα stabilization is also observed in human neuroblastoma SH-SY5Y cells, in which NOX4 expression is upregulated under hypoxic conditions. NOX4 knockdown inhibits glycolysis induced by hypoxia by preventing the activation of HIFα, the expression of glycolysis-related proteins (LDHA, and PDK1), as well as glucose uptake, lactate production, and ROS production [103]. Interestingly, in NOX4 knockout cancer cells, the expression of HIF1α-targeting genes, such as *SLC2A1*, encoding a glucose transporter, is prevented, thereby supporting the relevant role of NOX4-mediated metabolic reprogramming [104].

**Table 3:** NOX4 implication in cell metabolism regulation.

Cell Type/Tissue	Biological Effect	Cellular Effects	References
Non small lung cancer	GLUT1 expression ↑ LDHA expression ↑ PKM2 expression ↑	Glucose metabolism ↑ Glycolysis ↑ PPP ↑ Glutamine ↑ GSH production ↑	[98]
Glioblastoma specimens	NOX4 expression ↑ FOXM1 ↑ HIF1α ↑	Aerobic glycolysis ↑	[99]
Glioblastoma	GLUT1 ↑ HK2 expression ↑ LDHA expression ↑ PDK1 expression ↑	Survival ↑ Glycolysis ↑ EMT ↑ Proliferation ↑ Infiltration ↑	[102,105]
Neuroblastoma cell	NOX4 expression ↑ HIF1α ↑ LDHA expression ↑ PDK1 expression ↑ Glucose uptake ↑ Ki-67 expression ↑ PCNA expression ↑	Glycolysis ↑ Cell growth	[103]
Papillary thyroid cancer	mtROS generation ↑ HIF1α ↑ SLC2A1 ↑	Metabolic reprogramming Glycolysis ↑	[104]
Renal Carcinoma tissue and cells	NOX4 expression ↑ PKM2 expression ↑ mtROS ↑	Metabolic reprogramming Aerobic glycolysis ↑	[93]
Prostate cancer patients and cells	NOX4 expression ↑	Glycolysis ↑	[106]
Human aortic ECs	NOX4 expression↑ HIF1α ↑ PDK1 expression ↑	Glycolysis ↑ Hexosamine biosynthesis ↑ FAO ↑ Mitochondrial respiration ↓ Metabolic reprogramming	[107,108]
Cerebellar granule neuron precursors (CGNPs)	NOX4 expression↑ HIF1α ↑ Cyclin D2 expression ↑	Glycolysis ↑ Proliferation ↑	[109]
Neutrophil	[Lactate] ↑ PKM2 expression ↑	Glycolysis ↑ Warburg effect	[110]

GLUT1: glucose transporter 1; LDHA: lactate dehydrogenase A; PKM2: pyruvate kinase M2; PPP: pentose phosphate pathway; GSH: glutathione; FOXM1: Forkhead box M1; HIF1α: Hypoxic Inducible Factor 1 α; HK2: Hexokinase II; PDK1: pyruvate dehydrogenase kinase-1; EMT: Epithelial Mesenchymal Transition; mtROS: mitochondrial ROS.

NOX4 is overexpressed also in other cancer cells and tissues, including renal carcinoma cells [93]. In these cells, NOX4 localizes to the inner mitochondrial membrane and is allosterically regulated by adenosine triphosphate (ATP) levels, thus contributing to metabolic reprogramming [93]. During aerobic glycolysis, the depletion of mitochondrial ATP activates NOX4 activity that triggers an increase in mitochondrial ROS (mtROS). NOX4-mediated metabolic reprogramming including an increase in PKM2 expression, proving that NOX4 plays the key role of mitochondrial energetic sensor and fulfils the function of metabolic checkpoint, coupling the metabolic switch to cancer cell survival [93].

In the heart, NOX4 plays a protective role in the cardiac response to load-induced stress, involving an enhancement of myocardial capillary density and functional cardiac compensation. The molecular mechanism underlying the proangiogenic role of NOX4 includes NOX4-dependent ROS generation that, in turn, leads to enhanced HIF1 activation and an increased release of VEGF, which promotes capillarization [108]. NOX4-overexpression also promotes the metabolic reprogramming of glucose and fatty acid metabolism, by enhancing the activity of hexosamine biosynthetic pathways, reducing glucose oxidation by TCA cycle and increasing fatty acid oxidation, thus facilitating cardiac adaptation to chronic stress [108].

In physiopathological processes, such as the regulation of vascular functions, NOX4-derived ROS regulate HIF1α stabilization [111]. In endothelial cells, HIF1 induces an increase in the expression of glycolytic enzymes and of PDK1, which reduces mitochondrial respiratory capacity. Therefore, NOX4-derived ROS dynamically regulate endothelial metabolic plasticity and, consequently, endothelial activation and vascular health [107].

Cerebellar development is another physiological process in which NOX4 plays a crucial role. Here, NOX4-derived ROS promote HIF1α stabilization that, in turn, triggers the transcription of target genes related with a glycolytic phenotype. In cerebellar granule neuron precursors, NOX4-mediated HIF1α activation is also involved in the regulation of cell proliferation, by promoting cyclin D2 accumulation [109].

Neutrophil extracellular traps (NETs) play a crucial role in the physiological innate immune defense. NETosis induces a marked NOX4-dependent increase in ECAR, LDH activity, PKM2 dimerization, and a reduction in PKM2 activity, promoting lactate formation through the Warburg effect [110].

## 6. Modulation of NOX Family Expression as Therapeutic Target to Control Cell Metabolism

NOX-dependent ROS generation plays a significant role in the development of several malignancies, pathologies, and disease [112,113]. However, many studies have described the involvement of NOXs in the modulation of cell metabolism in several experimental systems.

Therapeutic approaches based on the application of generic antioxidant molecules in human trials have attracted much attention, but specific pharmacological strategies that selectively target oxidative pathways are still lacking [114]. Therefore, since NOX homologs are mainly regulated by enhanced expression and/or activation mechanisms, modulation of NOXs expression, as well as their activity, are considered new promising therapeutic approaches. For instance, NOX suppression by chemical inhibition or genetic silencing impacts on cancer cells leading to a decrease in glycolysis, a loss of cell viability, and an inhibition of cancer growth in vivo [31]. Consistently, mice deficient in NOX2 (NOX2KO) show a smaller visceral adipose deposit, attenuated visceral adipocyte hypertrophy, and diminished visceral adipose macrophage infiltration compared with wild-type mice. In NOX2KO mice, the glucose regulation was improved and detrimental effects were attenuated, in response to a high fat diet [115].

The therapeutic role of different miRNAs in the regulation of NOX expression has been explored in several studies. For instance, miR-146a shows a full alignment of 11-nucletide within the 3′-UTR of the human NOX4 and attenuates upregulation of NOX4 expression induced by glucose or thrombin in a diabetic atherothrombosis mouse model [116]. Similarly, miR-25 shows a 7-nucleotide match within 3′-UTR of NOX4 [117]. In high glucose-treated glomerular mesangial cells, the decreased level of miR-25 correlates with increased NOX4 expression [117]. NOX4 is also a direct target of miR-423-5p, both in vitro and in vivo, and the reduced expression levels of miR-423-5p correlates with enhanced NOX4 expression [118].

Further therapeutic approaches with miRNAs are based on the targeting of the cytosolic regulatory subunits of NOXs. For instance, miR-126 overexpression decreased NOX2 activation by reducing the expression of p67^phox^ and Rac family small GTPase 1 [119].

Currently, no FDA-approved miRNA-related drugs are available. The main limitation of this therapeutic approach is selectivity in targeting a NOX isoform in a specific single cell and/or tissue in a controlled manner. To date, the majority of miRNA-based therapeutic approaches have been focused on NOX4 modulation. Therefore, further studies are required to identify new miRNAs able to selectively modulate the other NOX homologs.

In conclusion, the interplay of NOX-modulated redox signaling pathways associated with metabolism is novel and is still far from being fully elucidated. Therefore, a better understanding of how NOX enzymes drive these complex adaptive responses will provide new mechanistic insights into metabolic reprogramming that may contribute to the development of new molecular and more selective therapeutic strategies.

## Figures and Tables

**Table 1:** NOX1 implication in cell metabolism regulation.

Cell Type/Tissue	Biological Effect	Cellular Effects	References
Pancreatic Cancer specimens/Colon cancer cells	NOX1 expression ↑ [NAD^+^] ↑ HK2 activity ↑	Glycolysis ↑	[31]
C2C12 cells	p70S6k ↑	Glycolysis ↑	[47]
Hepatic cancer cells	UTP-glucose-1-phosphate uridylyltransferase expression ↓ PEPCK expression ↑ GDH expression ↓ AST expression ↑ GMP reductase 2 expression ↑ cytosolic HMG-CoA synthase expression ↑	Glycolysis ↑ Reprogramming of glutamine metabolisms Nucleotide synthesis ↑ Lipid, steroid and isoprenoid biosynthesis ↑	[32]

HK2: Hexokinase II; PEPCK: phosphoenolpyruvate carboxykinase; GDH: glutamate dehydrogenase; AST: aspartate aminotransferase; HMG-CoA synthase: hydroxymethylglutaryl-CoA synthase.

**Table 2:** NOX2 implication in cell metabolism regulation.

Cell Type/Tissue	Biological Effect	Cellular Effects	References
M1 macrophages	HIF1α stabilization ↑ PDK1 activity ↑ PDH activity ↓ [Lactate] ↑	Metabolic reprogramming	[58,59]
Ovarian Cancer cells	HIF1α ↑ GLUT1 expression ↑ HK2 expression ↑	Glycolysis ↑ Metabolic reprogramming	[60]
RW264.7 monocyte/macrophage-like cells	NOX2 expression ↑ HIF1α ↑ GLUT1 expression ↑ HK2 activity ↑	Atherosclerotic microenvironment Glycolysis ↑	[61]
acute myeloid leukemia	Glucose uptake ↑ UCP2 expression ↑ AMPK phosphorylation ↑ PFKFB3 expression ↑	Glycolysis ↑ Sphingolipid metabolism ↑ Fatty acid oxidation ↑ Purine metabolism ↑	[62,63]
Glioblastoma multiforme and glioma cell	NOX2 activity ↑ Glucose uptake ↑ HK2 activity ↑	Glycolysis ↑	[64]
neutrophils	PFK-2 activity ↑	Glycolysis ↑	[65]

HIF1α: Hypoxic Inducible Factor 1 α; PDK1: pyruvate dehydrogenase kinase 1; PDH: pyruvate dehydrogenase; HK2: Hexokinase II; UCP2: uncoupling protein 2; PFKFB3: 6-phosphofructo-2-kinase/fructose-2,6-bisphosphatase; PFK-2: phosphofructo-2-kinase.

## Data Availability

The data presented in this study are available in the Reference list herein reported.
